# A Case of Fibrolamellar Hepatocellular Carcinoma in Which Tumor Control Was Achieved by Re‐Administering Atezolizumab and Bevacizumab

**DOI:** 10.1002/cnr2.70090

**Published:** 2024-12-19

**Authors:** Satoru Hagiwara, Itsuki Oda, Kazuomi Ueshima, Yoriaki Komeda, Naoshi Nishida, Akihiro Yoshida, Tomoki Yamamoto, Naoya Omaru, Takuya Matsubara, Masatoshi Kudo

**Affiliations:** ^1^ Department of Gastroenterology and Hepatology Kindai University Faculty of Medicine Osaka Japan; ^2^ Department of Clinical Genetics Kindai University Faculty of Medicine Osaka Japan

**Keywords:** atezolizumab, bevacizumab, combination therapy, fibrolamellar hepatocellular carcinoma, radiation therapy

## Abstract

**Background:**

Fibrolamellar hepatocellular carcinoma (FL‐HCC) clinically occurs in young people aged 20–30 years, who often have a normal liver background. We propose a treatment for such cases in which a combination therapy of atezolizumab and bevacizumab is followed by sandwiching radiation therapy to release tumor antigens and then re‐administering the combination therapy of atezolizumab and bevacizumab (ABC conversion therapy).

**Case:**

The patient is a 15‐year‐old girl. On April 18, 2022, she noticed skin yellowing and visited her local doctor. Computed tomography (CT) revealed a large mass in the right lobe of the liver and bile duct obstruction due to the tumor. She also had a nodule on her chest that appeared to be a metastatic tumor and was referred to Kinki University Hospital in April 2023. She was suspected to have FL‐HCC based on contrast‐enhanced ultrasound and CT scan results. There were findings suggestive of lung metastasis; however, she underwent a right hepatic lobectomy on May 17, 2023, considering the risk of liver failure and intra‐abdominal bleeding due to the large liver tumor. A CT scan conducted on July 25, 2022, showing increased lung metastases, and she started atezolizumab/bevacizumab combination treatment on October 20, 2022. On March 15, 2023, multiple lung metastases and new intrahepatic lesions appeared, which was diagnosed as progressive disease (PD), and lenvatinib was discontinued. On November 17, 2023, radiation therapy (25 Gy/5 Fr) was administered to the lung and intrahepatic lesions to release tumor antigens, and on November 27, 2023, atezolizumab and bevacizumab combination treatment was resumed to control the tumor.

**Conclusion:**

Combination therapy with atezolizumab, bevacizumab, and radiation therapy may be an option for the treatment of FL‐HCC.

## 1 Introduction

Fibrolamellar hepatocellular carcinoma (FL‐HCC), first reported by Edmondson in 1956 [1], is a disease common in Caucasians in Europe and America but extremely rare in Japan [2]. Clinically, it affects people aged 20–30 years, who often have normal liver function [2]. Furthermore, FL‐HCC is generally resistant to immune checkpoint inhibitor (ICI) treatment. It has been reported that this is due to low immunogenicity resulting from low tumor mutational burden (TMB) and negative Programmed death‐ligand 1 (PD L1) expression status [3, 4]. We proposed a treatment for such cases in which a combination therapy of atezolizumab and bevacizumab is followed by sandwiching transcatheter arterial chemoembolization (TACE) to release tumor antigens and then re‐administering the combination therapy of atezolizumab and bevacizumab (ABC conversion therapy) [5]. HCC had invaded the bile ducts; therefore, TACE could not be performed, but tumor control was achieved by sandwiching radiation therapy to the liver and lung lesions as an alternative treatment for tumor antigen release. Combination therapy with atezolizumab, bevacizumab, and radiation therapy may be a treatment option for patients with FL‐HCC.

## 2 Case

A 15‐year‐old female with no notable medical history complained of yellowing of the skin. There was no relevant family history. She visited a local doctor with fever and cold symptoms on April 11, 2022, and was treated for COVID‐19 infection using oral medications. On April 18, 2022, she noticed yellowing of her skin after an illness and revisited the doctor. Computed tomography (CT) revealed a large mass in the right lobe of the liver and bile duct obstruction due to the tumor. A nodule that appeared to be a metastatic tumor was also observed in the chest. A large liver tumor and lung metastasis were suggested at a young age, and thorough examination and treatment at a specialized facility were recommended. The patient was referred to Kindai University Hospital in April 2023.

### 2.1 Symptoms at Referral

Height: 150.8 cm; weight: 38.4 kg; body temperature: 36.3°C; blood pressure: 96/60 mmHg; pulse: 99 beats/min.

Respiratory rate: 16 breaths/min. Clear consciousness, no palpebral conjunctival anemia, yellowing of the bulbar conjunctiva, and a flat/soft abdomen.

The liver was palpable with two transverse fingers at the midline, and the spleen was not palpable.

### 2.2 Oral Medication at Referral

None.

### 2.3 Post‐Referral Blood Test

Elevated hepatobiliary enzymes and bilirubin levels were also observed (Table 1). The albumin value slightly decreased; no increase was observed in the ammonia value. A marked increase in protein induced by vitamin K absence‐II levels was also observed. Hepatitis virus markers were negative for HBV and HCV.

**TABLE 1:** Laboratory data at referral.

Hematology	
WBC	5.19/μL
RBC	406 × 10^4^/μL
Hb	11.9 g/dL
Hct	37.4%
PLT	24.8 × 10^4^/μL
Neutro	68.8%
Lympho	17.0%
Eosino	3.1%

Coagulation	
PT	95.9%
INR	1.02

Blood chemistry	
TP	7.6 g/dL
Alb	3.6 g/dL
BUN	8 mg/dL
Cr	0.46 mg/dL
T‐Bil	8.2 mg/dL
D‐Bil	6.3 mg/dL
ALP	351 U/L
LDH	180 U/L
AST	87 U/L
ALT	137 U/L
γGTP	161 U/L
NH_3_	38 μmol/L
BNP	6.8 ng/mL

Viral marker	
HBsAg	(−)
HBcAb	(−)
HBsAb	(−)
HCVAb	(−)

Tumor marker	
AFP	6 ng/mL
PIVKAII	3494 mAU/mL
CEA	0.2 ng/mL
CA19‐9	51 U/mL

Abbreviations: AFP, alpha‐fetoprotein; ALT, alanine aminotransferase; AST, aspartate aminotransferase; BNP, brain natriuretic hormone; CA19‐9, carbohydrate antigen 19‐9; CEA, carcinoembryonic antigen; HBcAb, hepatitis B core antibody; HBsAb, hepatitis B surface antibody; HBsAg, hepatitis B surface antigen; HCVAb, hepatitis C virus antibody; INR, international normalized ratio; PIVKAII, prothrombin inhibitor produced by vitamin K deficiency; PLT, platelet; PT, prothrombin time.

### 2.4 Contrast‐Enhanced CT After Referral

**FIGURE 1:**
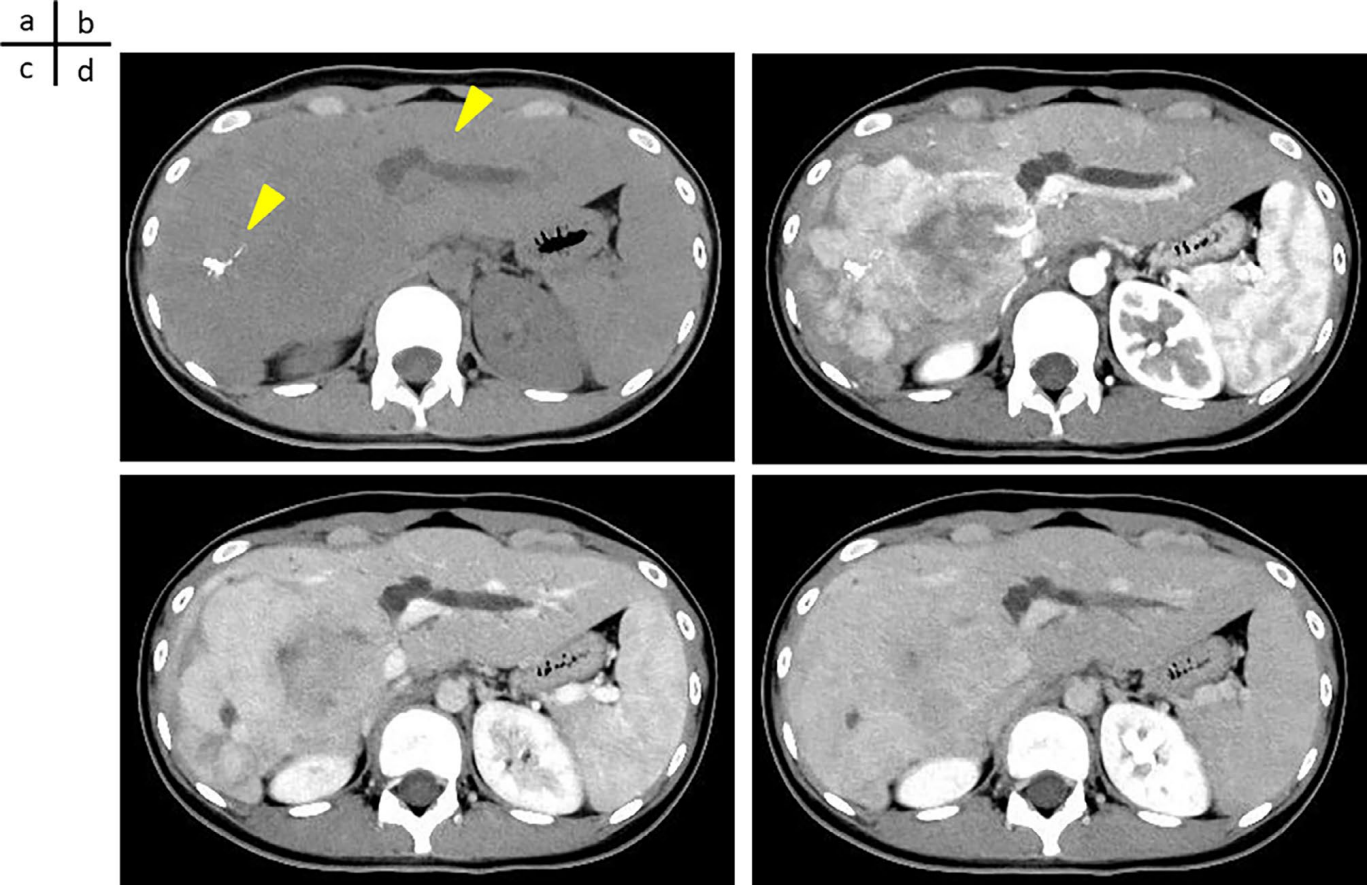
Contrast CT examination after referral. (a) Plain CT revealed a mass with a major diameter of 107 mm in the right lobe of the liver, with a slightly lower absorption than the liver parenchyma. Calcification is observed inside the tumor (arrow). Dilation of the left intrahepatic bile duct is observed (arrow). (b) In the arterial phase, most masses show heterogeneous deep staining, but the masses on the hepatic portal side show gradually increasing dark staining. The margin of the tumor is multilobed. (c) Staining persists even in the portal venous phase. (d) Staining persists even in the equilibrium phase. In addition, a nodule suspected of metastasis is observed in the left lung (data not shown).

Plain CT revealed a mass with a major diameter of 107 mm in the right lobe of the liver, which showed slightly lower absorption than the liver parenchyma. Calcification is observed inside the tumor (Figure 1a). In the arterial phase, most masses showed heterogeneous deep staining; however, the masses on the hepatic portal side showed gradually increasing dark staining. The tumor margins were multilobed (Figure 1b). Staining persisted even during the portal venous phase (Figure 1c). Staining persisted even in the equilibrium phase. In addition, a nodule suspected to be a metastasis was observed in the left lung (data not shown) (Figure 1d).

### 2.5 Contrast‐Enhanced Abdominal Ultrasound Examination After Referral

**FIGURE 2:**
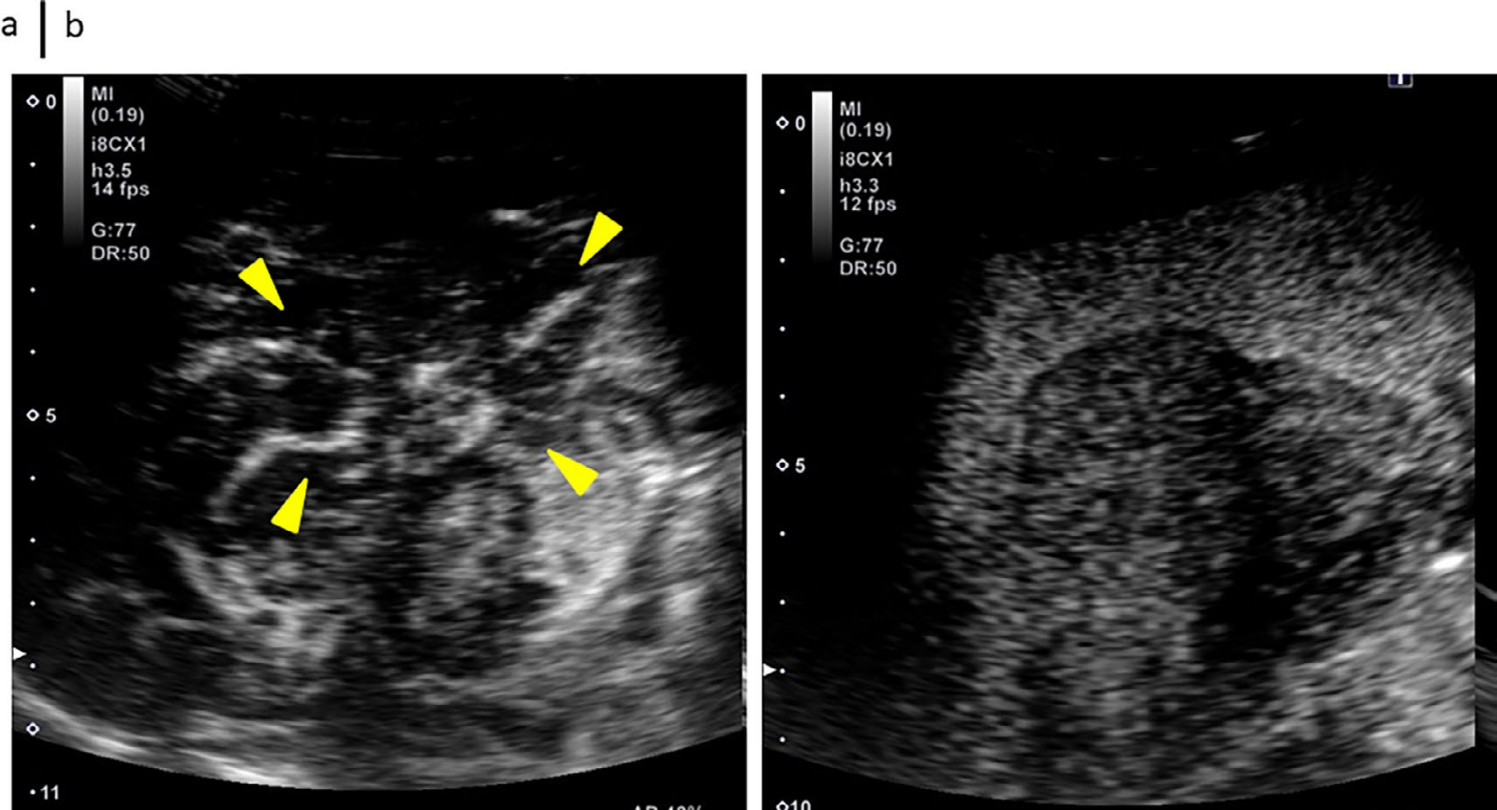
Contrast‐enhanced abdominal ultrasound examination after referral. (a) In the arterial‐dominated phase, it is extracted as a hypervascular mass, with some axle‐shaped blood vessel structures also observed (arrow). (b) In the Kupffer phase, it is extracted as a defect image.

A hypervascular mass was detected in the arterial‐dominated phase, and axle‐shaped blood vessels were observed (Figure 2a). In the Kupffer phase, it was extracted as a defect image (Figure 2b).

### 2.6 Gadolinium Ethoxybenzyl Diethylenetriamine Pentaacetic Acid‐Enhanced Magnetic Resonance Imaging (EOB‐MRI) After Referral

**FIGURE 3:**
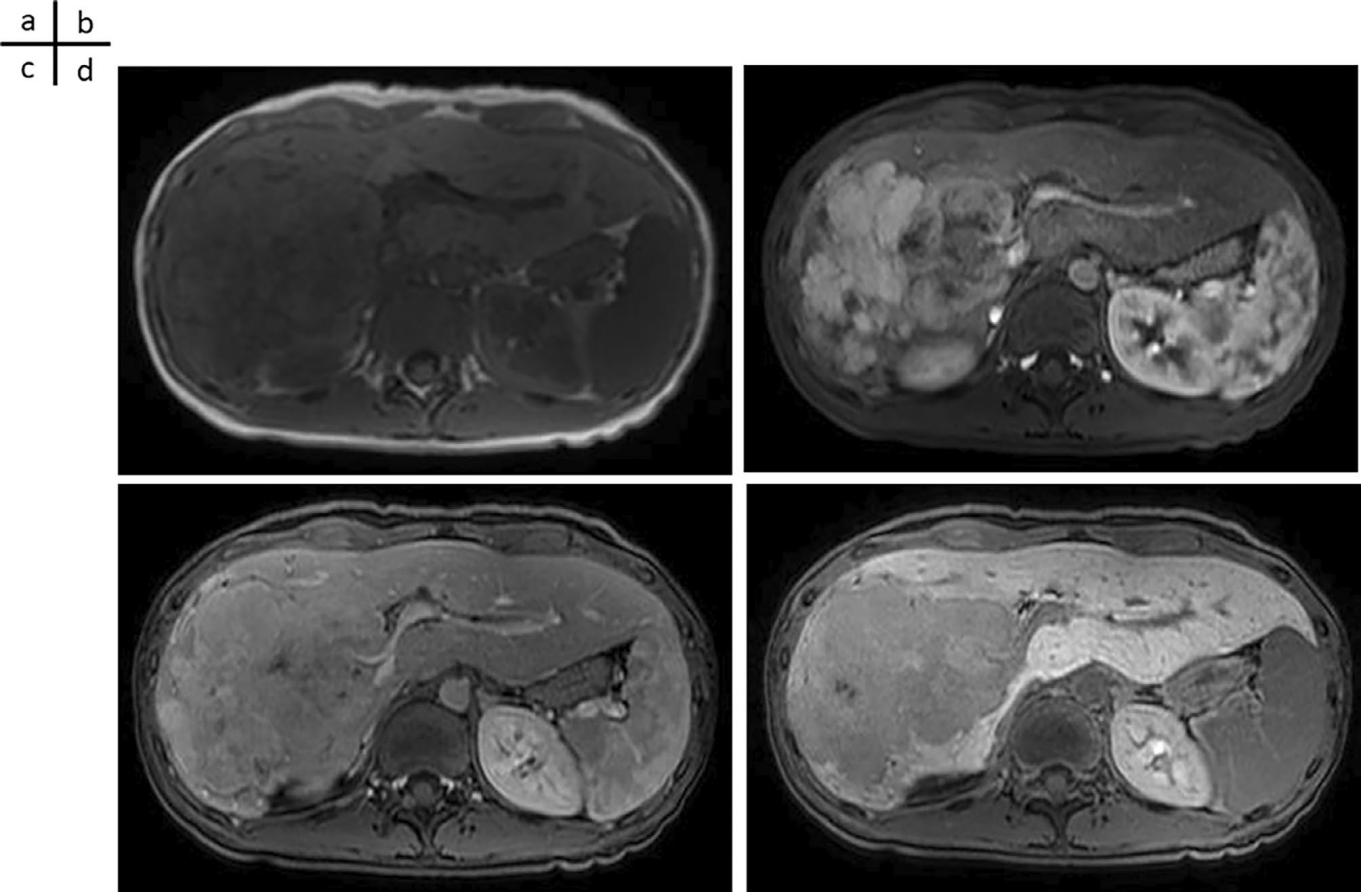
EOB‐MRI examination after introduction. (a) On plain MRI (T2WI), the mass exhibits a slightly lower density than the liver parenchyma. (b) In the arterial phase, most masses show heterogeneous deep staining; however, the masses on the hepatic portal side show gradually increasing dark staining. The tumor margin is multilobed. (c) Staining persists even during the portal venous phase. (d) In the hepatocyte phase, the mass exhibits decreased uptake of EOB.

Plain MRI (T2WI) showed that the mass had a slightly lower density than the liver parenchyma (Figure 3a). In the arterial phase, most masses showed heterogeneous deep staining; however, the masses on the hepatic portal side showed gradually increasing dark staining (Figure 3b). The tumor margins were multilobed. Staining persisted even during the portal venous phase (Figure 3c). In the hepatocyte phase, the mass exhibited decreased EOB (Figure 3d).

### 2.7 Magnetic Resonance Cholangiopancreatography (MRCP) After Referral

**FIGURE 4:**
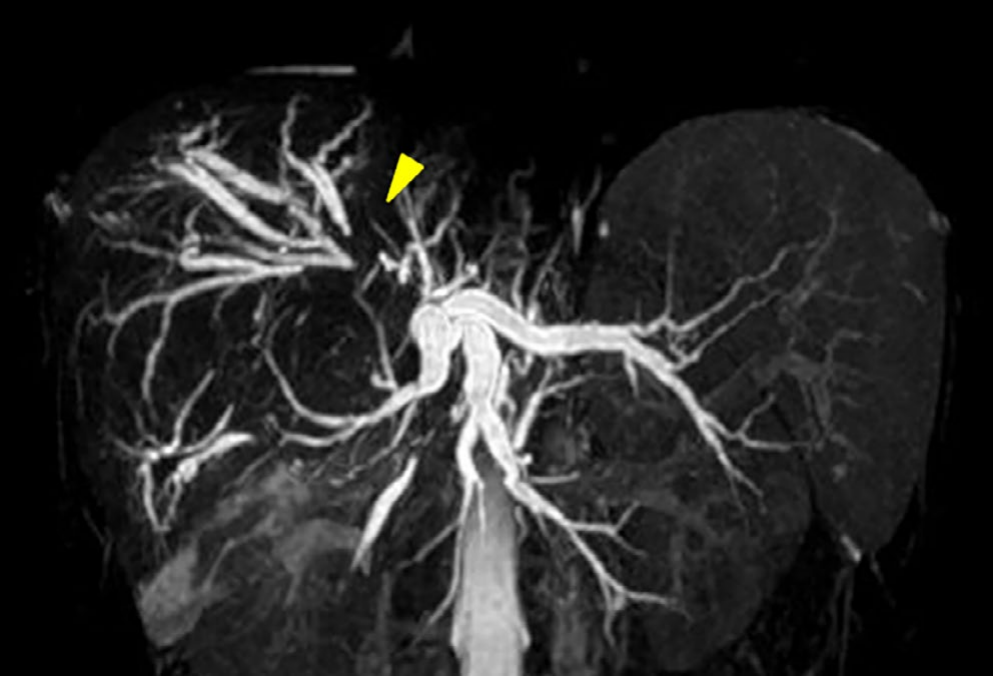
MRCP examination after referral. Owing to the mass displacement in the right lobe of the liver, the hilar bile duct is narrowed, and the left and right intrahepatic bile ducts are dilated (arrows).

The hilar bile duct was narrowed owing to the mass displacement in the right lobe of the liver, and the left and right intrahepatic bile ducts were dilated (Figure 4).

### 2.8 Macroscopic Findings of the Resected Specimen

**FIGURE 5:**
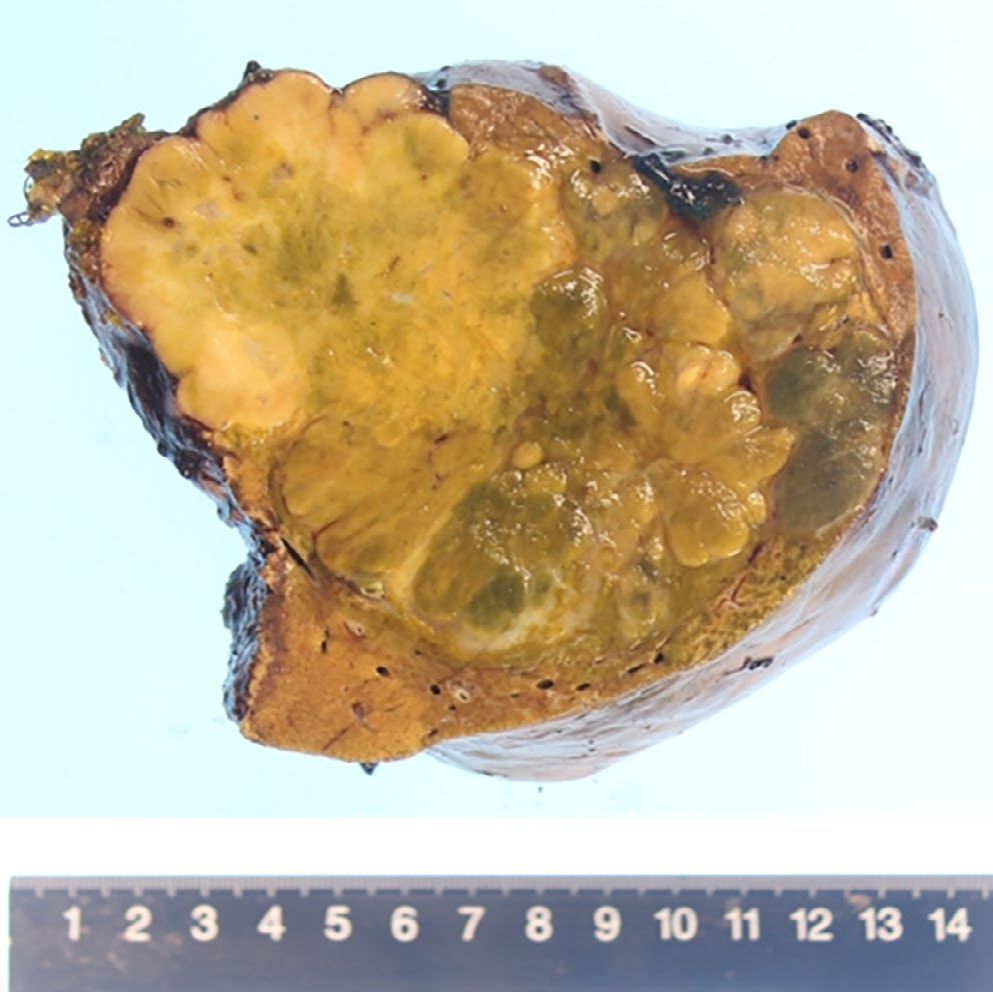
Macroscopic findings of resected specimen. The long axis is approximately 13 cm, and macroscopically, it appeared as a multinodular, fused, and solid tumor with lobulated margins. It also has coatings in certain areas. No evident central scarring was observed.

The major axis was approximately 13 cm, and macroscopically, it was a multinodular, fused, solid tumor with lobulated tumor margins. It also had coatings in certain areas (Figure 5). No evident central scarring was observed.

### 2.9 Histopathology Findings

**FIGURE 6:**
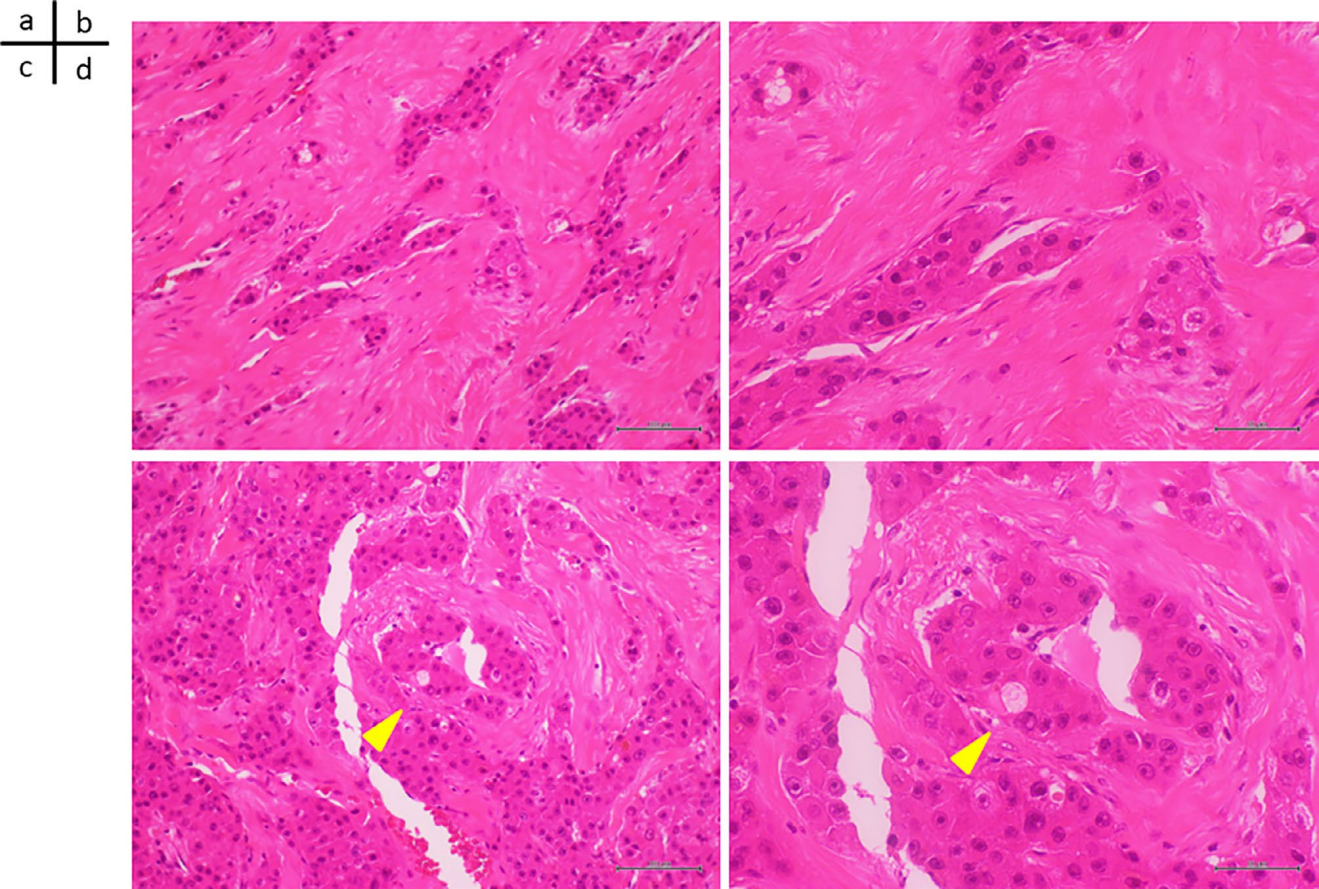
Histopathological findings. (a) H&E staining (magnification ×10) and (b) H&E staining (magnification ×20) shows atypical cells with round nuclei with clear nucleoli and abundant eosinophilic cells. Thick hyalinized fiber bundles are embedded in these atypical cells in a layered manner, and tumor cells proliferate as cables or small masses. (c) H&E staining (magnification ×10) and (d) H&E staining (magnification ×20) shows small vacuoles (pale bodies) exhibiting a sand grain‐like inclusion structure within the cytoplasmic bodies of tumor cells (arrows). The final pathological diagnosis was fibrolamellar carcinoma of the liver, Va1, Vv0, Vp0, b0, sm‐(4 mm), with positive lymph node metastasis.

Hematoxylin and eosin (H&E) staining (low‐power image) (Figure 6a) and H&E staining (high‐power image) showed a round nucleus with a clear nucleolus and abundant eosinophilic cells (Figure 6b). Atypical cells were also observed. Thick hyalinized fiber bundles were embedded in layers in these atypical cells, and the tumor cells proliferated as cables or small masses. In H&E staining (low‐power image) (Figure 6c) and H&E staining (high‐power image), small vacuoles (pale bodies) exhibiting a sand grain‐like inclusion structure were observed within the cytoplasmic bodies of tumor cells (Figure 6d). The final pathological diagnosis was fibrolamellar carcinoma (FLC) of the liver, Va1, Vv0, Vp0, b0, and sm (4 mm), with positive lymph node metastasis.

### 2.10 Immunohistology Findings

**FIGURE 7:**
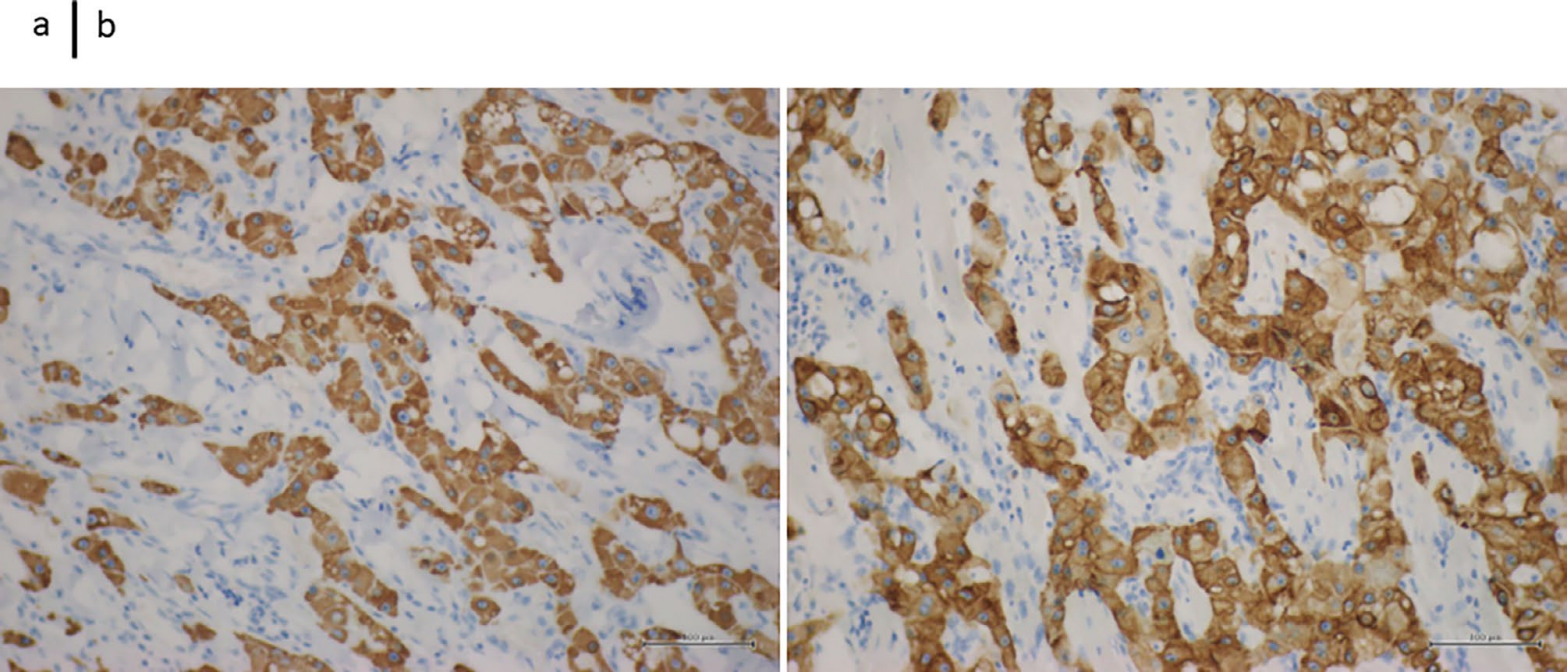
Immunohistology findings. (a) Immunostaining shows positive HepPar1 findings (magnification ×10). (b) Immunostaining also showed positive findings for CK7 (magnification ×10).

Immunostaining showed a positive finding for hepatocyte paraffin 1 (HepPar1). Immunostaining also revealed positivity for CK7 (Figure 7a).

### 2.11 Genetic Test Results

Using the liver resection specimen from May 17, 2023, we investigated using the FoundationOneCDx Cancer Genome Profile. The tumor content was 54.1%. The main results of Foundation One were Microsatellite instability‐stable, TMB: 0Muts/Mb, and CD79A, MPL, MSH2, Notch Receptor 3, SMARCA4 gene variants, and ERBB3 rearrangement. As for secondary findings, MSH2 and SMARCA4 gene variants were detected, but the MSH2 gene was evaluated as likely benign, and the SMARCA4 gene was evaluated as conflicting pathogenicity.

### 2.12 Plain Chest CT and Positron Emission Tomography (PET)‐CT Scan 1 Year and 5 Months After Presentation

**FIGURE 8:**
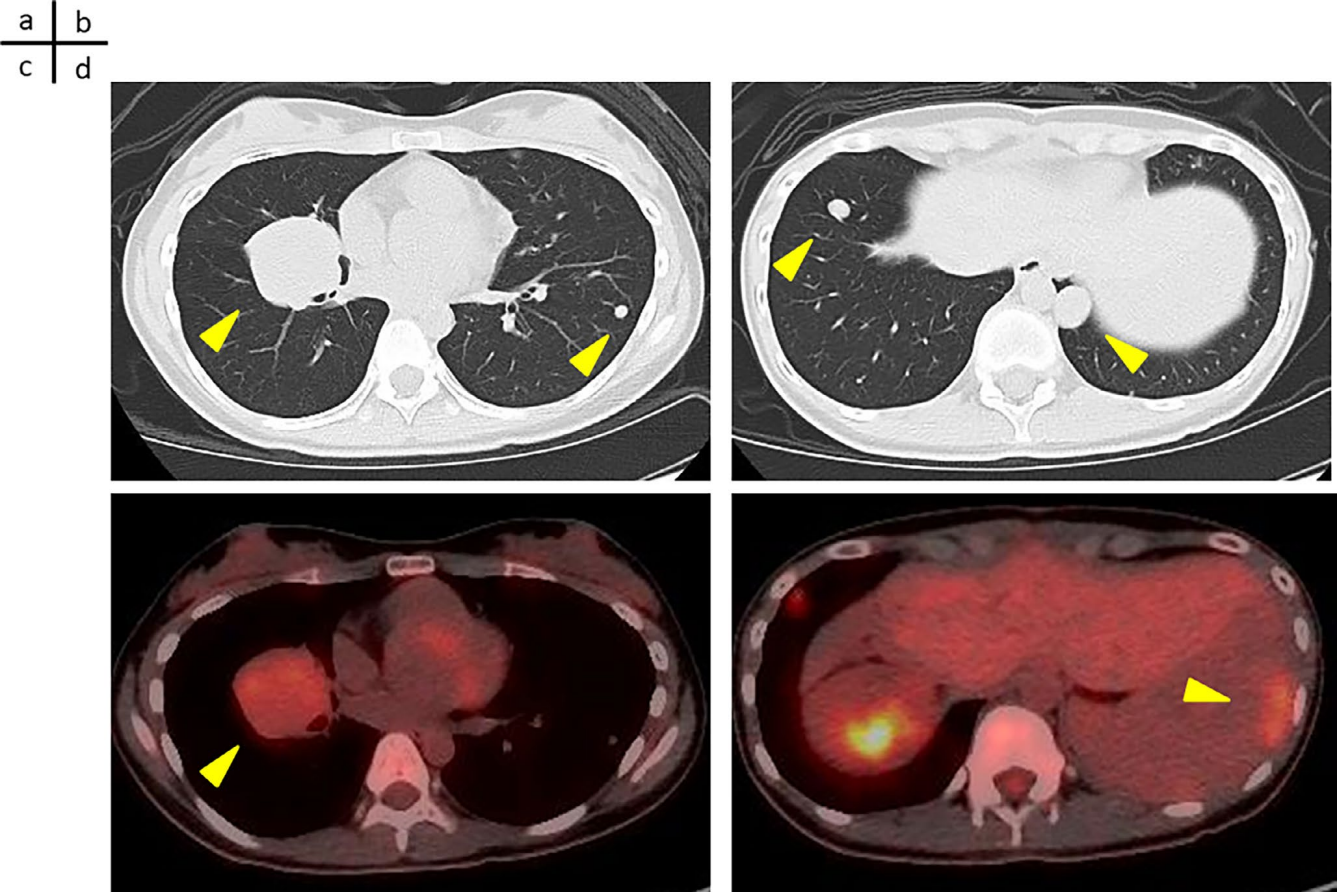
Plain chest CT scan and PET‐CT scan 1 year and 5 months after referral. (a) Plain chest CT showing a mass with a mild FGD accumulation near the right hilum (arrow). (b) Plain chest CT showing an enlarged nodule in the left lung at the time of presentation (arrow). (c) PET‐CT reveals a mass with a mild FGD accumulation near the right hilum (arrow). (d) PET‐CT showing peritoneal dissemination (arrow).

Plain chest CT revealed a mass with mild fluorine‐18 deoxyglucose (FGD) accumulation near the right hilum (Figure 8a). Plain chest CT revealed an enlarged nodule in the left lung observed at presentation (Figure 8b). PET‐CT reveals a mass with mild FGD accumulation near the right hilum (Figure 8c). PET‐CT showed peritoneal dissemination (Figure 8d).

### 2.13 Simple Chest CT Scan After Radiation Therapy

**FIGURE 9:**
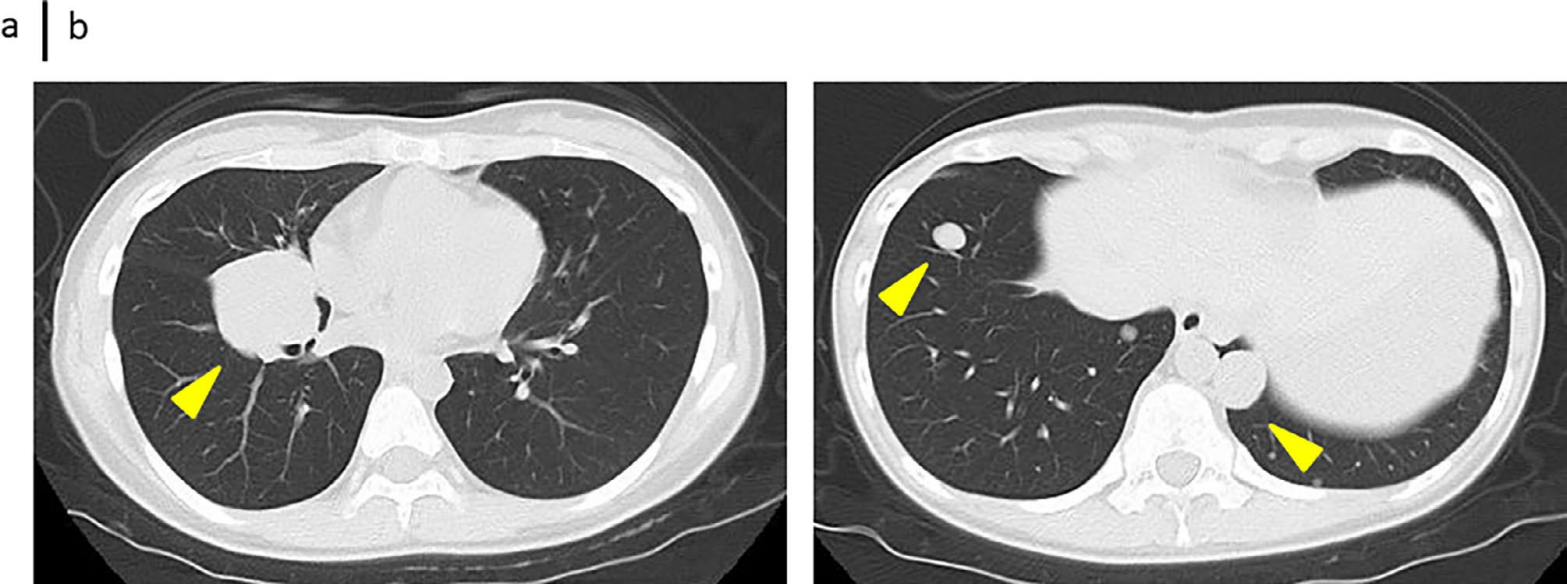
Simple chest CT examination after radiation therapy. (a) The tumor near the right lung hilum did not increase after treatment. (b) The mass in the left lung did not increase after treatment.

The tumor near the right hilum did not show any growth after treatment (Figure 9a). The mass in the left lung did not increase after treatment (Figure 9b).

### 2.14 Clinical Course After Referral

**FIGURE 10:**
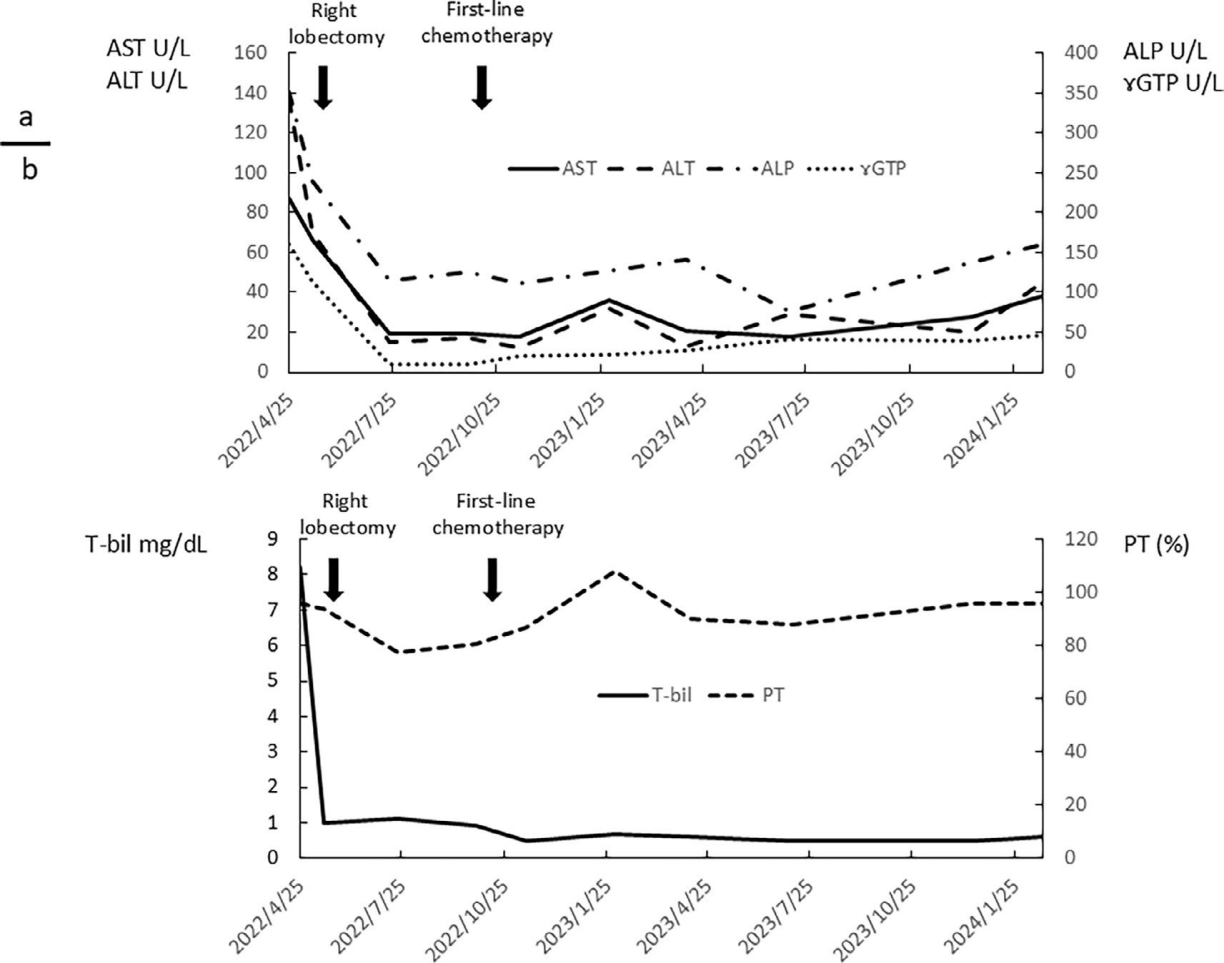
Clinical course of liver function. (a) Changes in AST, ALT, ALP, and ɤGTP. (b) Changes in T‐bil and PT.

The patient first visited our hospital on April 25, 2022, and underwent Endoscopic retrograde cholangiopancreatography on April 27, 2023, for obstructive jaundice. Hepatic hilar stenosis was caused by liver mass displacement, and a bile duct stent was inserted. Hepatocellular carcinoma was suspected based on the contrast‐enhanced US and CT scan results. A CT scan on July 25, 2022 showed increased lung metastases, and combination treatment with atezolizumab and bevacizumab was initiated on October 20, 2022, in accordance with Japanese treatment guidelines. On November 5, 2022, the patient developed strangulated ileus after HCC surgery and underwent laparoscopic ileus release surgery. Combination treatment with atezolizumab and bevacizumab was resumed on November 28, 2022; however, multiple lung metastases and new intrahepatic lesions appeared on March 15, 2023, and PD was discontinued. Durvalumab and tremelimumab combination therapy was initiated as a second‐line treatment on March 28, 2023. On April 18, 2023, the patient developed a fever and diarrhea. A diagnosis of immune‐related adverse event enteritis (G3) was made based on the lower endoscopy results, and prednisolone (PSL) administration at 30 mg/day was initiated. Enteritis persisted even after PSL administration; 180 mg of infliximab was added and enteritis improved. Lenvatinib 4 mg/day was initiated on August 16, 2023; however, peritoneal dissemination was observed on October 30, 2023, and PD was discontinued. On November 17, 2023, radiation therapy (25 Gy/5 Fr) was administered to the lung and intrahepatic lesions to release tumor antigens, and combination treatment with atezolizumab and bevacizumab was restarted on November 27, 2023. CT scan performed on January 29, 2024, showed that stable disease (SD) and tumor control were maintained. After restarting the atezolizumab/bevacizumab combination treatment, the patient continued treatment without any obvious adverse events. In addition, liver function remained stable after chemotherapy (Figure 10).

## 3 Discussion

FL‐HCC, first reported by Edmondson in 1956 [1], is a disease common in Caucasians in Europe and America but extremely rare in Japan [2]. Clinically, it occurs in young people aged 20–30 years, who often have a normal liver background [2]. The most common initial symptoms are abdominal pain and palpable mass, followed by abdominal distention, anorexia, fever, and jaundice [6]. However, FL‐HCC often forms a mass > 10 cm when discovered because there is no underlying liver disease and the onset is early [7]. The tumor, in this case, was > 10 cm, and the diagnosis was based on obstructive jaundice due to tumor growth. There are very few reports of its detection as a trigger for obstructive jaundice, and it is considered rare [8, 9]. However, approximately 20% of the initial FL‐HCC symptoms include jaundice [6]. Patients with FL‐HCC do not have any underlying liver disease, and bile duct obstruction is more likely to be a factor for jaundice than liver failure. There were findings suggestive of lung metastasis in this case; however, we performed an extended right hepatic lobectomy considering the risk of hepatic failure and intra‐abdominal hemorrhage, which would increase due to obstructive jaundice. Resection is beneficial even with a diagnosis of distant metastases. The 5‐year survival rate after resection in patients with distant metastases is reported to be 66%, with a median survival time of 34 months [2]. The high survival rate after resection is because there is no background liver disease, and the liver reserve is maintained, contributing to a prolonged prognosis.

Notably, various reports have been made regarding chemotherapy for patients with FL‐HCC. Kaseb et al. [10] reported that the overall survival of patients who received postoperative chemotherapy was 110.5 months and that combination therapy with interferon and 5‐fluorouracil was effective. Patt et al. [11] also reported that combination therapy with interferon and 5‐fluorouracil resulted in partial responses in five of eight patients. Furthermore, treatment with rapamycin, an mTOR inhibitor, has been reported to be effective [12]. Similarly, capecitabine, when administered as a second‐line therapy following sorafenib, has been associated with a 40% reduction in mortality risk compared to patients receiving best supportive care [13, 14]. In pediatric cases, a platinum‐based regimen resulted in 31% partial response; however, the 3‐year survival rate was only 22% [15]. There have been several reports as described above; however, the current situation is that no standard protocol has been established. Recently, ICIs have become the mainstream systemic chemotherapy for classical HCC [16]. Patients with FL‐HCC have also reported treatment results for ICI. There have been cases in which complete response (CR) was achieved with the combination of ipilimumab (anti‐CTLA4) and nivolumab (anti‐PD‐1) [17] and cases in which CR was achieved with triple combination chemoimmunotherapy of 5‐fluorouracil, interferon alfa‐2b, and nivolumab [18]. It was also reported that two Labian patients who received combination therapy with atezolizumab and bevacizumab showed no clinical efficacy [19]. Recent studies involving a relatively large number of cases have been reported [3, 4]. In 19 patients with FL‐HCC, partial responses were achieved in two of the 15 patients (13.3%) who received anti‐PD‐1 antibody inhibitor monotherapy. In addition, one of the four patients (25%) who received combination therapy with an anti‐PD‐1 antibody inhibitor and a cytotoxic T‐lymphocyte antigen‐4 (CTLA‐4) inhibitor achieved a partial response. A partial response was achieved in three (15.8%) of the 19 patients. The median progression‐free and overall survival were 5.5 and 26.0 months, respectively. The results of ICI treatment for patients with FL‐HCC have been poor in previous reports. This study's limited response to ICI treatment was due to low immunogenicity caused by low TMB and negative PD L1 expression status. In this case, combination therapy with atezolizumab and bevacizumab was administered but was not successful. The lack of success was thought to be due to low immunogenicity caused by low TMB in the FoundationOneCDx cancer gene panel test. Therefore, the intrahepatic mass and lung metastases were irradiated to release the tumor antigens, and combination therapy with atezolizumab and bevacizumab was repeated. We previously proposed a treatment in which combination therapy with atezolizumab and bevacizumab is followed by sandwiching TACE to release tumor antigens. The combination therapy with atezolizumab and bevacizumab (ABC conversion therapy) is then re‐administered [5]. These treatments have dramatically improved therapeutic effects. In this case, hepatocellular carcinoma had invaded the bile ducts; therefore, TACE could not be performed. However, as an alternative treatment for tumor antigen release, we sandwiched radiotherapy between the liver and lung lesions. Therefore, combination therapy with atezolizumab, bevacizumab, and radiation therapy may also be effective, and further accumulation of cases is required. In addition, clinical trials of new ICI and tyrosine kinase inhibitor combination therapies other than the combination of atezolizumab and bevacizumab are also underway, and the results are expected to be promising [20].

A fusion protein encoded by DNAJB1‐PRKACA has been identified in most FLC cases [21]. Engelholm et al. [22] used CRISPR/Cas9 to induce the formation of a DNAJB1‐PRKACA fusion gene in wild‐type mice to confirm the role of DNAJB1‐PRKACA. This mouse model produces tumors with characteristics similar to those of human FLC. Notably, another theory revolves around the Carney complex, where certain FLC cases lack the characteristic DNAJB1‐PRKACA fusion gene [23]. This indicates the existence of a molecular cause other than the DNAJB1‐PRKACA fusion gene for FLC formation. In addition to this fusion gene, activating promoter mutations in telomerase reverse transcriptase [24], mutations in mucin 415 [25] and BRAC2 [26], local deletions in 19p13.1 [26], excess long intergenic non‐protein coding RNA 473 and carbonic anhydrase 12 [27], and several other molecular and genetic changes in FLC have been reported. Furthermore, although studies in B‐cell lymphomas have been conducted, it has been suggested that mutations that result in retargeting of super‐enhancers are involved in the development of B‐cell lymphomas [28]. The presence or absence of the DNAJB1‐PRKACA fusion gene could not be investigated in this case; however, various genetic mutations were identified by FoundationOneCDx cancer gene panel testing. This is an essential finding when examining the carcinogenic factors associated with FL‐HCC.

In conclusion, we report an FL‐HCC case controlled by the re‐administration of atezolizumab and bevacizumab after radiation therapy. Therefore, combination therapy using atezolizumab and bevacizumab with radiation therapy may be an option for treating patients with ICI‐resistant FL‐HCC.

## Author Contributions

**Satoru Hagiwara:** conceptualization, data curation, writing – original draft, methodology, investigation. **Itsuki Oda:** writing – review and editing. **Kazuomi Ueshima:** writing – review and editing. **Yoriaki Komeda:** writing – review and editing. **Naoshi Nishida:** writing – review and editing. **Akihiro Yoshida:** writing – review and editing. **Tomoki Yamamoto:** writing – review and editing. **Naoya Omaru:** writing – review and editing. **Takuya Matsubara:** writing – review and editing. **Masatoshi Kudo:** writing – review and editing.

## Ethics Statement

The authors have nothing to report.

## Consent

Written consent was obtained from the patient to report the details of the case.

## Conflicts of Interest

The authors declare no conflicts of interest.

## Data Availability

The data that support the findings of this study are available on request from the corresponding author. The data are not publicly available due to privacy or ethical restrictions.
